# Bis(μ_2_-4-amino-3-nitro­benzoato)bis­(4-amino-3-nitro­benzoato)octa­butyldi-μ_3_-oxido-tetra­tin(IV)

**DOI:** 10.1107/S1600536810040146

**Published:** 2010-10-13

**Authors:** Yip-Foo Win, Chen-Shang Choong, Siang-Guan Teoh, Jia Hao Goh, Hoong-Kun Fun

**Affiliations:** a Department of Chemical Science, Faculty of Science, Universiti Tunku Abdul Rahman, 31900 Kampar, Perak, Malaysia; b School of Chemical Sciences, Universiti Sains Malaysia, 11800 USM, Penang, Malaysia; c X-ray Crystallography Unit, School of Physics, Universiti Sains Malaysia, 11800 USM, Penang, Malaysia

## Abstract

The tetranuclear molecules of the title compound, [Sn_4_(C_4_H_9_)_8_(C_7_H_5_N_2_O_4_)_4_O_2_], reside on a crystallographic inversion center. Both the two independent Sn atoms are five-coordinate, with distorted trigonal–bipyramidal geometries. One Sn atom is coordinated by two O atoms of the carboxyl­ate anions, one bridging O atom and two butyl groups and the other Sn atom is coordinated by an O atom of the carboxyl­ate anion, two bridging O atoms and two butyl groups. All the butyl groups are equatorial with respect to the SnO_3_ trigonal plane. The mol­ecular structure is stabilized by intra­molecular N—H⋯O hydrogen bonds. In the crystal, pairs of inter­molecular bifurcated acceptor N—H⋯O and C—H⋯O hydrogen bonds link the mol­ecules into chains along [10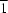]. Weak inter­molecular C—H⋯π and π–π inter­actions [centroid–centroid distance = 3.713 (2) Å] are also observed.

## Related literature

For general background to and applications of the title complex, see: Khoo & Hazell (1999 ▶); Parvez *et al.* (2004 ▶); Li *et al.* (2006 ▶); Win *et al.* (2008*a* ▶,*b* ▶). For closely related structures, see: Khoo & Hazell (1999 ▶); Parvez *et al.* (2004 ▶); Li *et al.* (2006 ▶); Win *et al.* (2008*b* ▶). For graph-set motifs, see: Bernstein *et al.* (1995 ▶). For the stability of the temperature controller used in the data collection, see: Cosier & Glazer (1986 ▶). 
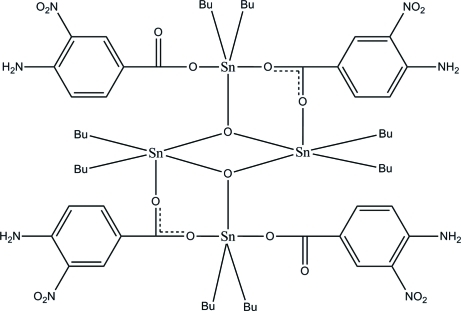


## Experimental

### 

#### Crystal data

[Sn_4_(C_4_H_9_)_8_(C_7_H_5_N_2_O_4_)_4_O_2_]
*M* *_r_* = 1688.18
Triclinic, 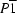

*a* = 11.9585 (9) Å
*b* = 13.0679 (10) Å
*c* = 13.1897 (10) Å
α = 76.256 (2)°
β = 67.445 (2)°
γ = 66.108 (2)°
*V* = 1732.1 (2) Å^3^
*Z* = 1
Mo *K*α radiation
μ = 1.50 mm^−1^
*T* = 100 K
0.20 × 0.15 × 0.06 mm

#### Data collection

Bruker APEXII DUO CCD area-detector diffractometer
Absorption correction: multi-scan (*SADABS*; Bruker, 2009 ▶) *T*_min_ = 0.758, *T*_max_ = 0.912
32184 measured reflections
11359 independent reflections
8603 reflections with *I* > 2σ(*I*)
*R*_int_ = 0.055

#### Refinement

*R*[*F* ^2^ > 2σ(*F* ^2^)] = 0.037
*wR*(*F* ^2^) = 0.116
*S* = 1.04
11359 reflections
410 parameters
H-atom parameters constrained
Δρ_max_ = 1.26 e Å^−3^
Δρ_min_ = −1.18 e Å^−3^

### 

Data collection: *APEX2* (Bruker, 2009 ▶); cell refinement: *SAINT* (Bruker, 2009 ▶); data reduction: *SAINT*; program(s) used to solve structure: *SHELXTL* (Sheldrick, 2008 ▶); program(s) used to refine structure: *SHELXTL*; molecular graphics: *SHELXTL*; software used to prepare material for publication: *SHELXTL* and *PLATON* (Spek, 2009 ▶).

## Supplementary Material

Crystal structure: contains datablocks global, I. DOI: 10.1107/S1600536810040146/fj2350sup1.cif

Structure factors: contains datablocks I. DOI: 10.1107/S1600536810040146/fj2350Isup2.hkl

Additional supplementary materials:  crystallographic information; 3D view; checkCIF report

## Figures and Tables

**Table 1:** Hydrogen-bond geometry (Å, °) *Cg*1 is the centroid of the C16–C21 phenyl ring.

*D*—H⋯*A*	*D*—H	H⋯*A*	*D*⋯*A*	*D*—H⋯*A*
N1—H1*N*1⋯O6	0.91	2.59	3.422 (4)	153
N1—H2*N*1⋯O1	1.03	1.83	2.644 (5)	133
N3—H1*N*3⋯O4^i^	0.88	2.04	2.910 (4)	167
N3—H2*N*3⋯O5	0.88	2.06	2.669 (4)	125
C17—H17*A*⋯O4^i^	0.93	2.51	3.246 (5)	137
C30—H30*A*⋯*Cg*1^ii^	0.96	2.80	3.584 (5)	139

Symmetry codes: (i) 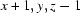; (ii) 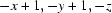.

**Fig. 1.**
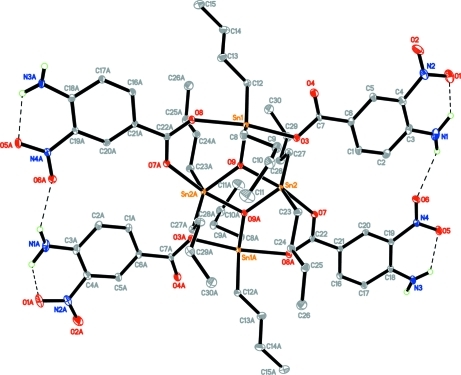
The molecular structure of the title complex, showing 30% probability displacement ellipsoids and the atom-numbering scheme. The suffix A corresponds to symmetry code [-x, -y+1, -z]. Intramolecular hydrogen bonds are shown as dashed lines and all C-bound H atoms were omitted for clarity.

**Fig. 2.**
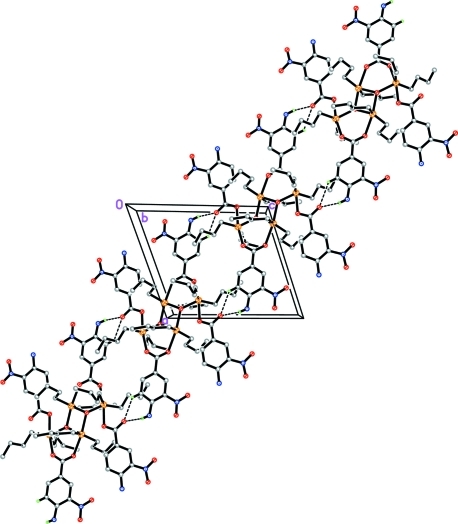
The crystal structure of the title complex, viewed along the b axis, showing a one-dimensional chain along the [101] direction. H atoms not involved in intermolecular hydrogen bonds (dashed lines) have been omitted for clarity.

## supplementary crystallographic information

### Comment

In general, there are many well-documented structures on complexes isolated from 1:1 molar ratio reaction between diorganotin(IV) with the respective organic acids (Khoo & Hazell, 1999; Parvez *et al.*, 2004; Li *et al.*, 2006; Win *et al.*, 2008*a*,*b*). This dimeric structure is known as organodistannoxane dimer with the core geometry consisting of a centrosymmetric planar Sn_2_O_2_ group (Win *et al.*, 2008*a*,*b*). The centrosymmetric planar Sn_2_O_2_ group is bonded to the exo- and endocyclic tin(IV) atom moiety *via* the bridging oxygen atoms so that the oxygen atoms are tri-coordinated (Khoo & Hazell, 1999; Parvez *et al.*, 2004; Li *et al.*, 2006). In this study, the crystal structure of the title complex is similar to bis(2,3-dibromopropionato)tetrabutyldistannoxane(IV) dimer and consists of a centrosymmetric planar Sn_2_O_2_ group (Win *et al.*, 2008*b*). The only exception is 4-amino-3-nitrobenzoic acid is utilized in the reaction to obtain the title complex.

The asymmetric unit of the title complex (Fig. 1) lies on a crystallographic inversion center and comprises of one-half molecule, with the other half of the molecule is generated by symmetry code -x, -y+1, -z. The Sn1 atom is five-coordinated by two butyl groups in equatorial position, an O atom of the monodentate carboxylate anion, an O atom of the bridging carboxylate atom and one bridging O atom in a distorted trigonal bipiramidal geometry. The Sn2 atom also has a distorted trigonal bipiramidal geometry, being coordinated by two butyl groups in equatorial position, one bridging carboxylate O atom and two bridging O atoms. Intramolecular N1—H2N1···O1 and N3—H2N3···O5 hydrogen bonds (Table 1) form two different six-membered rings, generating *S*(6) ring motifs (Bernstein *et al.*, 1995) which help to stabilize the molecular structure. All geometric parameters are consistent to those observed in closely related structures (Khoo & Hazell, 1999; Parvez *et al.*, 2004; Li *et al.*, 2006; Win *et al.*, 2008*b*).

In the crystal structure, pairs of intermolecular bifurcated acceptor N3—H1N3···O4 and C17—H17A···O4 hydrogen bonds (Table 1) link adjacent molecules into one-dimensional chains incorporating *R*^2^_1_(6) hydrogen bond ring motifs along the [101] direction (Bernstein *et al.*, 1995, Fig. 2). Further stabilization of the crystal structure is provided by weak intermolecular C30—H30A···*Cg*1 interactions (Table 1) as well as *Cg*1···*Cg*2 aromatic stacking interactions where *Cg*1 and *Cg* 2 are the centroids of the C16-C21 and C1-C6 benzene rings.

### Experimental

The title complex was obtained by heating under reflux in a 1:1 molar mixture of dibutyltin(IV) oxide (0.50 g, 2 mmol) and 4-amino-3-nitrobenzoic acid (0.36 g, 2 mmol) in methanol (50 ml) for 4 h. Clear yellowish solution was isolated by filtration and kept in a bottle. After 12 days, yellow single crystals (0.71 g, 75.8 % yeild) were collected. *M.p.* 525.8–527.4 K. Analysis found for C_60_H_92_N_8_O_18_Sn_4_: C, 42.84; H, 5.43; N, 6.49; Sn, 28.28 %. Calculated found for C_60_H_92_N_8_O_18_Sn_4_: C, 42.68; H, 5.49; N, 6.64; Sn, 28.13 %.

### Refinement

The amino group H atoms were located from the difference Fourier map and constrained to ride with the parent atom with *U*_iso_ = 1.2 *U*_eq_(N). All other H atoms were placed in their calculated positions, with C—H = 0.93 – 0.97 Å, and refined using a riding model with *U*_iso_ = 1.2 or 1.5 *U*_eq_(C). The rotating group model was used for the methyl groups. The highest residual electron density peak and the deepest hole were located at 0.72 Å from atom Sn1.

### Crystal data

**Table:**

[Sn_4_(C_4_H_9_)_8_(C_7_H_5_N_2_O_4_)_4_O_2_]	*Z* = 1
*M**_r_* = 1688.18	*F*(000) = 852
Triclinic, *P*1	*D*_x_ = 1.618 Mg m^−^^3^
Hall symbol: -P 1	Mo *K*α radiation, λ = 0.71073 Å
*a* = 11.9585 (9) Å	Cell parameters from 5645 reflections
*b* = 13.0679 (10) Å	θ = 3.4–33.2°
*c* = 13.1897 (10) Å	µ = 1.50 mm^−^^1^
α = 76.256 (2)°	*T* = 100 K
β = 67.445 (2)°	Block, yellow
γ = 66.108 (2)°	0.20 × 0.15 × 0.06 mm
*V* = 1732.1 (2) Å^3^	

### Data collection

**Table:**

Bruker APEXII DUO CCD area-detector diffractometer	11359 independent reflections
Radiation source: fine-focus sealed tube	8603 reflections with *I* > 2σ(*I*)
graphite	*R*_int_ = 0.055
φ and ω scans	θ_max_ = 31.5°, θ_min_ = 1.7°
Absorption correction: multi-scan (*SADABS*; Bruker, 2009)	*h* = −17→17
*T*_min_ = 0.758, *T*_max_ = 0.912	*k* = −19→18
32184 measured reflections	*l* = −19→19

### Refinement

**Table:**

Refinement on *F*^2^	Primary atom site location: structure-invariant direct methods
Least-squares matrix: full	Secondary atom site location: difference Fourier map
*R*[*F*^2^ > 2σ(*F*^2^)] = 0.037	Hydrogen site location: inferred from neighbouring sites
*wR*(*F*^2^) = 0.116	H-atom parameters constrained
*S* = 1.04	*w* = 1/[σ^2^(*F*_o_^2^) + (0.0611*P*)^2^] where *P* = (*F*_o_^2^ + 2*F*_c_^2^)/3
11359 reflections	(Δ/σ)_max_ = 0.001
410 parameters	Δρ_max_ = 1.25 e Å^−^^3^
0 restraints	Δρ_min_ = −1.17 e Å^−^^3^

### Special details

**Table:**

Experimental. The crystal was placed in the cold stream of an Oxford Cryosystems Cobra open-flow nitrogen cryostat (Cosier & Glazer, 1986) operating at 100.0 (1)K.
Geometry. All esds (except the esd in the dihedral angle between two l.s. planes) are estimated using the full covariance matrix. The cell esds are taken into account individually in the estimation of esds in distances, angles and torsion angles; correlations between esds in cell parameters are only used when they are defined by crystal symmetry. An approximate (isotropic) treatment of cell esds is used for estimating esds involving l.s. planes.
Refinement. Refinement of F^2^ against ALL reflections. The weighted R-factor wR and goodness of fit S are based on F^2^, conventional R-factors R are based on F, with F set to zero for negative F^2^. The threshold expression of F^2^ > 2sigma(F^2^) is used only for calculating R-factors(gt) etc. and is not relevant to the choice of reflections for refinement. R-factors based on F^2^ are statistically about twice as large as those based on F, and R- factors based on ALL data will be even larger.

### Fractional atomic coordinates and isotropic or equivalent isotropic displacement parameters (Å^2^)

**Table:**

	*x*	*y*	*z*	*U*_iso_*/*U*_eq_	
Sn1	−0.15407 (2)	0.426491 (18)	0.258267 (17)	0.01235 (6)	
Sn2	0.126384 (19)	0.388757 (17)	0.012249 (17)	0.01183 (6)	
O1	0.5285 (3)	−0.1013 (3)	0.4294 (3)	0.0452 (9)	
O2	0.3268 (4)	−0.0394 (3)	0.5298 (3)	0.0469 (9)	
O3	0.0372 (2)	0.29294 (19)	0.21639 (19)	0.0150 (5)	
O4	−0.0160 (2)	0.2104 (2)	0.3863 (2)	0.0230 (5)	
O5	0.9029 (2)	−0.0002 (2)	−0.1901 (2)	0.0233 (5)	
O6	0.7210 (3)	0.0470 (2)	−0.0558 (2)	0.0254 (6)	
O7	0.3299 (2)	0.3217 (2)	−0.10236 (19)	0.0183 (5)	
O8	−0.3316 (2)	0.5805 (2)	0.2675 (2)	0.0187 (5)	
O9	−0.0734 (2)	0.47928 (19)	0.09963 (18)	0.0133 (4)	
N1	0.5993 (3)	−0.0496 (3)	0.2134 (3)	0.0313 (8)	
H1N1	0.6573	−0.0435	0.1450	0.038*	
H2N1	0.6222	−0.0939	0.2830	0.038*	
N2	0.4123 (4)	−0.0430 (3)	0.4400 (3)	0.0315 (8)	
N3	0.9151 (3)	0.0899 (3)	−0.3964 (2)	0.0192 (6)	
H1N3	0.9467	0.1258	−0.4600	0.023*	
H2N3	0.9574	0.0317	−0.3594	0.023*	
N4	0.7854 (3)	0.0571 (2)	−0.1543 (2)	0.0173 (6)	
C1	0.2968 (3)	0.1390 (3)	0.1681 (3)	0.0186 (7)	
H1A	0.2693	0.1787	0.1084	0.022*	
C2	0.4240 (3)	0.0748 (3)	0.1495 (3)	0.0212 (7)	
H2A	0.4809	0.0718	0.0776	0.025*	
C3	0.4715 (3)	0.0126 (3)	0.2369 (3)	0.0213 (7)	
C4	0.3797 (4)	0.0193 (3)	0.3434 (3)	0.0211 (7)	
C5	0.2486 (3)	0.0864 (3)	0.3609 (3)	0.0182 (6)	
H5A	0.1902	0.0895	0.4322	0.022*	
C6	0.2058 (3)	0.1470 (3)	0.2745 (3)	0.0163 (6)	
C7	0.0658 (3)	0.2188 (3)	0.2964 (3)	0.0161 (6)	
C8	−0.2737 (3)	0.3386 (3)	0.2691 (3)	0.0190 (7)	
H8A	−0.3379	0.3446	0.3420	0.023*	
H8B	−0.3195	0.3771	0.2164	0.023*	
C9	−0.2096 (4)	0.2138 (3)	0.2494 (3)	0.0238 (7)	
H9A	−0.1820	0.1707	0.3118	0.029*	
H9B	−0.1334	0.2038	0.1843	0.029*	
C10	−0.3018 (4)	0.1683 (3)	0.2336 (4)	0.0283 (8)	
H10A	−0.2612	0.0875	0.2313	0.034*	
H10B	−0.3802	0.1828	0.2968	0.034*	
C11	−0.3378 (6)	0.2205 (5)	0.1287 (4)	0.0438 (12)	
H11A	−0.3958	0.1894	0.1240	0.066*	
H11B	−0.2611	0.2043	0.0655	0.066*	
H11C	−0.3793	0.3004	0.1307	0.066*	
C12	−0.1180 (3)	0.4888 (3)	0.3732 (3)	0.0191 (7)	
H12A	−0.0676	0.4252	0.4117	0.023*	
H12B	−0.0650	0.5344	0.3321	0.023*	
C13	−0.2355 (4)	0.5582 (4)	0.4589 (3)	0.0248 (8)	
H13A	−0.2862	0.6223	0.4211	0.030*	
H13B	−0.2886	0.5128	0.5010	0.030*	
C14	−0.2041 (4)	0.6002 (4)	0.5377 (3)	0.0280 (8)	
H14A	−0.1521	0.6466	0.4963	0.034*	
H14B	−0.1533	0.5365	0.5758	0.034*	
C15	−0.3241 (5)	0.6682 (4)	0.6222 (4)	0.0352 (10)	
H15A	−0.2992	0.6927	0.6707	0.053*	
H15B	−0.3754	0.6224	0.6644	0.053*	
H15C	−0.3737	0.7325	0.5851	0.053*	
C16	0.5905 (3)	0.2917 (3)	−0.3655 (3)	0.0166 (6)	
H16A	0.5465	0.3451	−0.4107	0.020*	
C17	0.7173 (3)	0.2297 (3)	−0.4104 (3)	0.0178 (6)	
H17A	0.7574	0.2407	−0.4859	0.021*	
C18	0.7904 (3)	0.1483 (3)	−0.3449 (3)	0.0152 (6)	
C19	0.7236 (3)	0.1349 (3)	−0.2307 (3)	0.0135 (6)	
C20	0.5918 (3)	0.1990 (3)	−0.1861 (3)	0.0133 (6)	
H20A	0.5499	0.1885	−0.1109	0.016*	
C21	0.5243 (3)	0.2764 (3)	−0.2514 (3)	0.0136 (6)	
C22	0.3840 (3)	0.3441 (3)	−0.2034 (3)	0.0138 (6)	
C23	0.0916 (4)	0.2564 (3)	−0.0232 (3)	0.0194 (7)	
H23A	0.1537	0.1853	−0.0055	0.023*	
H23B	0.0061	0.2570	0.0241	0.023*	
C24	0.1005 (3)	0.2635 (3)	−0.1432 (3)	0.0183 (6)	
H24A	0.0363	0.3332	−0.1604	0.022*	
H24B	0.1850	0.2651	−0.1908	0.022*	
C25	0.0794 (4)	0.1652 (3)	−0.1674 (3)	0.0215 (7)	
H25A	0.1367	0.0952	−0.1421	0.026*	
H25B	−0.0088	0.1689	−0.1266	0.026*	
C26	0.1044 (4)	0.1659 (4)	−0.2894 (4)	0.0301 (9)	
H26A	0.1004	0.0983	−0.3020	0.045*	
H26B	0.1885	0.1701	−0.3309	0.045*	
H26C	0.0400	0.2299	−0.3125	0.045*	
C27	0.2107 (3)	0.4357 (3)	0.1018 (3)	0.0174 (6)	
H27A	0.1436	0.4668	0.1682	0.021*	
H27B	0.2737	0.3685	0.1245	0.021*	
C28	0.2768 (4)	0.5210 (3)	0.0386 (3)	0.0202 (7)	
H28A	0.3422	0.4917	−0.0292	0.024*	
H28B	0.2134	0.5900	0.0189	0.024*	
C29	0.3397 (3)	0.5474 (3)	0.1050 (3)	0.0221 (7)	
H29A	0.3960	0.4773	0.1313	0.027*	
H29B	0.3931	0.5906	0.0567	0.027*	
C30	0.2445 (4)	0.6125 (4)	0.2031 (3)	0.0269 (8)	
H30A	0.2908	0.6194	0.2452	0.040*	
H30B	0.1869	0.5733	0.2487	0.040*	
H30C	0.1957	0.6860	0.1774	0.040*	

### Atomic displacement parameters (Å^2^)

**Table:**

	*U*^11^	*U*^22^	*U*^33^	*U*^12^	*U*^13^	*U*^23^
Sn1	0.01042 (10)	0.01296 (11)	0.01006 (10)	−0.00221 (7)	−0.00232 (7)	0.00013 (8)
Sn2	0.01123 (10)	0.01097 (10)	0.01065 (10)	−0.00180 (7)	−0.00337 (7)	−0.00046 (7)
O1	0.0303 (17)	0.047 (2)	0.053 (2)	0.0028 (14)	−0.0281 (16)	−0.0003 (17)
O2	0.042 (2)	0.059 (2)	0.0247 (16)	−0.0027 (16)	−0.0162 (15)	0.0049 (15)
O3	0.0112 (10)	0.0149 (11)	0.0138 (10)	−0.0025 (8)	−0.0021 (8)	0.0003 (9)
O4	0.0167 (12)	0.0256 (14)	0.0165 (12)	−0.0040 (9)	−0.0023 (10)	0.0045 (10)
O5	0.0134 (11)	0.0237 (13)	0.0247 (13)	0.0002 (9)	−0.0071 (10)	0.0013 (10)
O6	0.0214 (13)	0.0294 (15)	0.0150 (12)	−0.0009 (10)	−0.0053 (10)	0.0010 (10)
O7	0.0117 (11)	0.0233 (13)	0.0119 (10)	−0.0004 (9)	−0.0021 (9)	−0.0007 (9)
O8	0.0135 (11)	0.0199 (12)	0.0174 (11)	0.0010 (9)	−0.0068 (9)	−0.0017 (9)
O9	0.0116 (10)	0.0127 (10)	0.0117 (10)	−0.0025 (8)	−0.0029 (8)	0.0009 (8)
N1	0.0133 (14)	0.0299 (18)	0.043 (2)	−0.0003 (12)	−0.0082 (14)	−0.0053 (16)
N2	0.0276 (18)	0.0309 (19)	0.0340 (19)	−0.0016 (13)	−0.0198 (15)	0.0013 (15)
N3	0.0104 (12)	0.0188 (14)	0.0184 (14)	−0.0006 (10)	−0.0018 (10)	0.0026 (11)
N4	0.0149 (13)	0.0170 (14)	0.0190 (14)	−0.0021 (10)	−0.0088 (11)	−0.0010 (11)
C1	0.0141 (15)	0.0159 (15)	0.0233 (17)	−0.0039 (11)	−0.0057 (13)	−0.0005 (13)
C2	0.0146 (15)	0.0217 (17)	0.0237 (17)	−0.0053 (12)	−0.0027 (13)	−0.0040 (14)
C3	0.0149 (15)	0.0163 (16)	0.0331 (19)	−0.0041 (12)	−0.0078 (14)	−0.0057 (14)
C4	0.0245 (18)	0.0179 (16)	0.0251 (17)	−0.0081 (13)	−0.0140 (14)	0.0015 (14)
C5	0.0148 (15)	0.0182 (16)	0.0206 (16)	−0.0051 (11)	−0.0073 (13)	0.0014 (13)
C6	0.0118 (14)	0.0142 (15)	0.0217 (16)	−0.0041 (11)	−0.0051 (12)	−0.0011 (12)
C7	0.0144 (14)	0.0132 (15)	0.0188 (15)	−0.0019 (11)	−0.0089 (12)	0.0025 (12)
C8	0.0149 (15)	0.0174 (16)	0.0218 (16)	−0.0056 (11)	−0.0040 (13)	−0.0006 (13)
C9	0.0226 (17)	0.0192 (17)	0.0300 (19)	−0.0075 (13)	−0.0078 (15)	−0.0041 (15)
C10	0.033 (2)	0.0203 (18)	0.034 (2)	−0.0134 (15)	−0.0121 (17)	0.0024 (16)
C11	0.066 (4)	0.041 (3)	0.047 (3)	−0.034 (3)	−0.031 (3)	0.004 (2)
C12	0.0168 (15)	0.0209 (17)	0.0175 (15)	−0.0020 (12)	−0.0073 (12)	−0.0038 (13)
C13	0.0201 (17)	0.033 (2)	0.0234 (18)	−0.0076 (14)	−0.0060 (14)	−0.0104 (16)
C14	0.035 (2)	0.033 (2)	0.0184 (17)	−0.0131 (16)	−0.0098 (16)	−0.0030 (15)
C15	0.043 (3)	0.035 (2)	0.026 (2)	−0.0060 (18)	−0.0123 (19)	−0.0121 (18)
C16	0.0147 (14)	0.0166 (15)	0.0148 (14)	−0.0032 (11)	−0.0048 (12)	0.0011 (12)
C17	0.0132 (14)	0.0192 (16)	0.0143 (14)	−0.0013 (11)	−0.0035 (12)	0.0003 (12)
C18	0.0118 (13)	0.0148 (15)	0.0173 (15)	−0.0048 (11)	−0.0034 (12)	−0.0007 (12)
C19	0.0121 (13)	0.0130 (14)	0.0159 (14)	−0.0039 (10)	−0.0060 (11)	−0.0007 (11)
C20	0.0124 (13)	0.0141 (14)	0.0122 (13)	−0.0048 (10)	−0.0026 (11)	−0.0014 (11)
C21	0.0102 (13)	0.0144 (14)	0.0117 (13)	−0.0014 (10)	−0.0012 (11)	−0.0026 (11)
C22	0.0132 (14)	0.0122 (14)	0.0168 (14)	−0.0028 (10)	−0.0061 (11)	−0.0038 (12)
C23	0.0211 (16)	0.0192 (16)	0.0171 (15)	−0.0079 (12)	−0.0042 (13)	−0.0024 (13)
C24	0.0202 (16)	0.0174 (16)	0.0195 (16)	−0.0063 (12)	−0.0090 (13)	−0.0020 (13)
C25	0.0188 (16)	0.0197 (17)	0.0275 (18)	−0.0053 (12)	−0.0076 (14)	−0.0073 (14)
C26	0.026 (2)	0.035 (2)	0.034 (2)	−0.0072 (15)	−0.0130 (17)	−0.0120 (18)
C27	0.0165 (15)	0.0186 (16)	0.0169 (15)	−0.0079 (12)	−0.0040 (12)	−0.0006 (12)
C28	0.0233 (17)	0.0228 (17)	0.0164 (15)	−0.0126 (13)	−0.0053 (13)	0.0007 (13)
C29	0.0185 (16)	0.0252 (18)	0.0259 (18)	−0.0125 (13)	−0.0081 (14)	0.0020 (14)
C30	0.0252 (19)	0.029 (2)	0.033 (2)	−0.0119 (15)	−0.0110 (16)	−0.0075 (17)

### Geometric parameters (Å, °)

**Table:**

Sn1—O9	2.022 (2)	C11—H11C	0.9600
Sn1—C8	2.117 (4)	C12—C13	1.520 (5)
Sn1—C12	2.125 (3)	C12—H12A	0.9700
Sn1—O3	2.200 (2)	C12—H12B	0.9700
Sn1—O8	2.243 (2)	C13—C14	1.504 (5)
Sn2—O9^i^	2.044 (2)	C13—H13A	0.9700
Sn2—C23	2.121 (3)	C13—H13B	0.9700
Sn2—C27	2.133 (3)	C14—C15	1.517 (6)
Sn2—O9	2.163 (2)	C14—H14A	0.9700
Sn2—O7	2.249 (2)	C14—H14B	0.9700
Sn2—Sn2^i^	3.2982 (4)	C15—H15A	0.9600
O1—N2	1.255 (5)	C15—H15B	0.9600
O2—N2	1.228 (5)	C15—H15C	0.9600
O3—C7	1.301 (4)	C16—C17	1.360 (4)
O4—C7	1.229 (4)	C16—C21	1.415 (4)
O5—N4	1.251 (4)	C16—H16A	0.9300
O6—N4	1.238 (4)	C17—C18	1.425 (5)
O7—C22	1.260 (4)	C17—H17A	0.9300
O8—C22^i^	1.260 (4)	C18—C19	1.415 (5)
O9—Sn2^i^	2.044 (2)	C19—C20	1.409 (4)
N1—C3	1.357 (5)	C20—C21	1.369 (5)
N1—H1N1	0.9127	C20—H20A	0.9300
N1—H2N1	1.0326	C21—C22	1.499 (4)
N2—C4	1.439 (5)	C22—O8^i^	1.260 (4)
N3—C18	1.348 (4)	C23—C24	1.527 (5)
N3—H1N3	0.8828	C23—H23A	0.9700
N3—H2N3	0.8795	C23—H23B	0.9700
N4—C19	1.439 (4)	C24—C25	1.527 (5)
C1—C2	1.363 (5)	C24—H24A	0.9700
C1—C6	1.404 (5)	C24—H24B	0.9700
C1—H1A	0.9300	C25—C26	1.518 (6)
C2—C3	1.413 (6)	C25—H25A	0.9700
C2—H2A	0.9300	C25—H25B	0.9700
C3—C4	1.408 (5)	C26—H26A	0.9600
C4—C5	1.408 (5)	C26—H26B	0.9600
C5—C6	1.371 (5)	C26—H26C	0.9600
C5—H5A	0.9300	C27—C28	1.526 (5)
C6—C7	1.500 (5)	C27—H27A	0.9700
C8—C9	1.533 (5)	C27—H27B	0.9700
C8—H8A	0.9700	C28—C29	1.521 (5)
C8—H8B	0.9700	C28—H28A	0.9700
C9—C10	1.543 (6)	C28—H28B	0.9700
C9—H9A	0.9700	C29—C30	1.517 (5)
C9—H9B	0.9700	C29—H29A	0.9700
C10—C11	1.528 (7)	C29—H29B	0.9700
C10—H10A	0.9700	C30—H30A	0.9600
C10—H10B	0.9700	C30—H30B	0.9600
C11—H11A	0.9600	C30—H30C	0.9600
C11—H11B	0.9600		
			
O9—Sn1—C8	110.89 (12)	C13—C12—H12B	108.1
O9—Sn1—C12	113.25 (12)	Sn1—C12—H12B	108.1
C8—Sn1—C12	135.33 (14)	H12A—C12—H12B	107.3
O9—Sn1—O3	78.94 (9)	C14—C13—C12	114.5 (3)
C8—Sn1—O3	100.88 (11)	C14—C13—H13A	108.6
C12—Sn1—O3	93.91 (11)	C12—C13—H13A	108.6
O9—Sn1—O8	89.41 (9)	C14—C13—H13B	108.6
C8—Sn1—O8	84.97 (12)	C12—C13—H13B	108.6
C12—Sn1—O8	88.89 (11)	H13A—C13—H13B	107.6
O3—Sn1—O8	168.19 (9)	C13—C14—C15	112.6 (4)
O9^i^—Sn2—C23	104.84 (12)	C13—C14—H14A	109.1
O9^i^—Sn2—C27	108.28 (12)	C15—C14—H14A	109.1
C23—Sn2—C27	146.36 (14)	C13—C14—H14B	109.1
O9^i^—Sn2—O9	76.78 (9)	C15—C14—H14B	109.1
C23—Sn2—O9	95.98 (11)	H14A—C14—H14B	107.8
C27—Sn2—O9	97.07 (11)	C14—C15—H15A	109.5
O9^i^—Sn2—O7	92.75 (9)	C14—C15—H15B	109.5
C23—Sn2—O7	87.89 (12)	H15A—C15—H15B	109.5
C27—Sn2—O7	84.87 (11)	C14—C15—H15C	109.5
O9—Sn2—O7	169.46 (9)	H15A—C15—H15C	109.5
O9^i^—Sn2—Sn2^i^	39.68 (6)	H15B—C15—H15C	109.5
C23—Sn2—Sn2^i^	103.12 (10)	C17—C16—C21	121.3 (3)
C27—Sn2—Sn2^i^	105.97 (10)	C17—C16—H16A	119.4
O9—Sn2—Sn2^i^	37.10 (6)	C21—C16—H16A	119.4
O7—Sn2—Sn2^i^	132.42 (6)	C16—C17—C18	121.9 (3)
C7—O3—Sn1	116.89 (19)	C16—C17—H17A	119.1
C22—O7—Sn2	133.4 (2)	C18—C17—H17A	119.1
C22^i^—O8—Sn1	139.1 (2)	N3—C18—C19	125.8 (3)
Sn1—O9—Sn2^i^	137.06 (11)	N3—C18—C17	117.9 (3)
Sn1—O9—Sn2	119.61 (11)	C19—C18—C17	116.3 (3)
Sn2^i^—O9—Sn2	103.22 (9)	C20—C19—C18	120.9 (3)
C3—N1—H1N1	121.9	C20—C19—N4	116.6 (3)
C3—N1—H2N1	112.1	C18—C19—N4	122.5 (3)
H1N1—N1—H2N1	125.5	C21—C20—C19	121.3 (3)
O2—N2—O1	121.7 (4)	C21—C20—H20A	119.4
O2—N2—C4	119.9 (3)	C19—C20—H20A	119.4
O1—N2—C4	118.4 (4)	C20—C21—C16	118.3 (3)
C18—N3—H1N3	111.7	C20—C21—C22	121.0 (3)
C18—N3—H2N3	118.7	C16—C21—C22	120.7 (3)
H1N3—N3—H2N3	128.4	O7—C22—O8^i^	126.0 (3)
O6—N4—O5	121.8 (3)	O7—C22—C21	117.0 (3)
O6—N4—C19	119.5 (3)	O8^i^—C22—C21	117.0 (3)
O5—N4—C19	118.7 (3)	C24—C23—Sn2	114.1 (2)
C2—C1—C6	122.2 (4)	C24—C23—H23A	108.7
C2—C1—H1A	118.9	Sn2—C23—H23A	108.7
C6—C1—H1A	118.9	C24—C23—H23B	108.7
C1—C2—C3	121.5 (3)	Sn2—C23—H23B	108.7
C1—C2—H2A	119.3	H23A—C23—H23B	107.6
C3—C2—H2A	119.3	C25—C24—C23	113.0 (3)
N1—C3—C4	124.9 (4)	C25—C24—H24A	109.0
N1—C3—C2	118.9 (4)	C23—C24—H24A	109.0
C4—C3—C2	116.2 (3)	C25—C24—H24B	109.0
C3—C4—C5	121.4 (4)	C23—C24—H24B	109.0
C3—C4—N2	122.7 (3)	H24A—C24—H24B	107.8
C5—C4—N2	115.9 (3)	C26—C25—C24	112.3 (3)
C6—C5—C4	121.0 (3)	C26—C25—H25A	109.1
C6—C5—H5A	119.5	C24—C25—H25A	109.1
C4—C5—H5A	119.5	C26—C25—H25B	109.1
C5—C6—C1	117.7 (3)	C24—C25—H25B	109.1
C5—C6—C7	119.6 (3)	H25A—C25—H25B	107.9
C1—C6—C7	122.7 (3)	C25—C26—H26A	109.5
O4—C7—O3	122.2 (3)	C25—C26—H26B	109.5
O4—C7—C6	121.6 (3)	H26A—C26—H26B	109.5
O3—C7—C6	116.2 (3)	C25—C26—H26C	109.5
C9—C8—Sn1	118.1 (2)	H26A—C26—H26C	109.5
C9—C8—H8A	107.8	H26B—C26—H26C	109.5
Sn1—C8—H8A	107.8	C28—C27—Sn2	115.0 (2)
C9—C8—H8B	107.8	C28—C27—H27A	108.5
Sn1—C8—H8B	107.8	Sn2—C27—H27A	108.5
H8A—C8—H8B	107.1	C28—C27—H27B	108.5
C8—C9—C10	112.1 (3)	Sn2—C27—H27B	108.5
C8—C9—H9A	109.2	H27A—C27—H27B	107.5
C10—C9—H9A	109.2	C29—C28—C27	112.5 (3)
C8—C9—H9B	109.2	C29—C28—H28A	109.1
C10—C9—H9B	109.2	C27—C28—H28A	109.1
H9A—C9—H9B	107.9	C29—C28—H28B	109.1
C11—C10—C9	113.5 (4)	C27—C28—H28B	109.1
C11—C10—H10A	108.9	H28A—C28—H28B	107.8
C9—C10—H10A	108.9	C30—C29—C28	114.2 (3)
C11—C10—H10B	108.9	C30—C29—H29A	108.7
C9—C10—H10B	108.9	C28—C29—H29A	108.7
H10A—C10—H10B	107.7	C30—C29—H29B	108.7
C10—C11—H11A	109.5	C28—C29—H29B	108.7
C10—C11—H11B	109.5	H29A—C29—H29B	107.6
H11A—C11—H11B	109.5	C29—C30—H30A	109.5
C10—C11—H11C	109.5	C29—C30—H30B	109.5
H11A—C11—H11C	109.5	H30A—C30—H30B	109.5
H11B—C11—H11C	109.5	C29—C30—H30C	109.5
C13—C12—Sn1	116.8 (2)	H30A—C30—H30C	109.5
C13—C12—H12A	108.1	H30B—C30—H30C	109.5
Sn1—C12—H12A	108.1		
			
O9—Sn1—O3—C7	173.3 (3)	C5—C6—C7—O3	164.7 (3)
C8—Sn1—O3—C7	−77.3 (3)	C1—C6—C7—O3	−15.1 (5)
C12—Sn1—O3—C7	60.4 (3)	O9—Sn1—C8—C9	83.0 (3)
O8—Sn1—O3—C7	163.8 (4)	C12—Sn1—C8—C9	−106.3 (3)
O9^i^—Sn2—O7—C22	−11.5 (3)	O3—Sn1—C8—C9	0.8 (3)
C23—Sn2—O7—C22	93.3 (3)	O8—Sn1—C8—C9	170.4 (3)
C27—Sn2—O7—C22	−119.6 (3)	Sn1—C8—C9—C10	−166.9 (3)
O9—Sn2—O7—C22	−18.5 (7)	C8—C9—C10—C11	66.1 (5)
Sn2^i^—Sn2—O7—C22	−12.6 (4)	O9—Sn1—C12—C13	121.2 (3)
O9—Sn1—O8—C22^i^	17.1 (4)	C8—Sn1—C12—C13	−49.4 (4)
C8—Sn1—O8—C22^i^	−94.0 (4)	O3—Sn1—C12—C13	−159.2 (3)
C12—Sn1—O8—C22^i^	130.3 (4)	O8—Sn1—C12—C13	32.3 (3)
O3—Sn1—O8—C22^i^	26.4 (7)	Sn1—C12—C13—C14	−179.8 (3)
C8—Sn1—O9—Sn2^i^	82.2 (2)	C12—C13—C14—C15	−179.6 (4)
C12—Sn1—O9—Sn2^i^	−90.8 (2)	C21—C16—C17—C18	−1.4 (5)
O3—Sn1—O9—Sn2^i^	179.71 (19)	C16—C17—C18—N3	179.4 (3)
O8—Sn1—O9—Sn2^i^	−2.23 (18)	C16—C17—C18—C19	0.4 (5)
C8—Sn1—O9—Sn2	−102.47 (15)	N3—C18—C19—C20	−178.5 (3)
C12—Sn1—O9—Sn2	84.59 (15)	C17—C18—C19—C20	0.4 (5)
O3—Sn1—O9—Sn2	−4.93 (11)	N3—C18—C19—N4	2.1 (5)
O8—Sn1—O9—Sn2	173.13 (13)	C17—C18—C19—N4	−179.1 (3)
O9^i^—Sn2—O9—Sn1	−176.76 (19)	O6—N4—C19—C20	2.5 (5)
C23—Sn2—O9—Sn1	79.36 (15)	O5—N4—C19—C20	−178.9 (3)
C27—Sn2—O9—Sn1	−69.56 (15)	O6—N4—C19—C18	−178.0 (3)
O7—Sn2—O9—Sn1	−169.5 (4)	O5—N4—C19—C18	0.6 (5)
Sn2^i^—Sn2—O9—Sn1	−176.76 (19)	C18—C19—C20—C21	−0.3 (5)
O9^i^—Sn2—O9—Sn2^i^	0.001 (2)	N4—C19—C20—C21	179.2 (3)
C23—Sn2—O9—Sn2^i^	−103.88 (13)	C19—C20—C21—C16	−0.6 (5)
C27—Sn2—O9—Sn2^i^	107.20 (13)	C19—C20—C21—C22	179.9 (3)
O7—Sn2—O9—Sn2^i^	7.2 (5)	C17—C16—C21—C20	1.4 (5)
C6—C1—C2—C3	−0.1 (6)	C17—C16—C21—C22	−179.0 (3)
C1—C2—C3—N1	−179.8 (4)	Sn2—O7—C22—O8^i^	3.3 (5)
C1—C2—C3—C4	1.1 (5)	Sn2—O7—C22—C21	−178.0 (2)
N1—C3—C4—C5	179.8 (4)	C20—C21—C22—O7	−4.1 (5)
C2—C3—C4—C5	−1.3 (5)	C16—C21—C22—O7	176.3 (3)
N1—C3—C4—N2	−1.7 (6)	C20—C21—C22—O8^i^	174.7 (3)
C2—C3—C4—N2	177.3 (3)	C16—C21—C22—O8^i^	−4.9 (5)
O2—N2—C4—C3	−175.4 (4)	O9^i^—Sn2—C23—C24	27.4 (3)
O1—N2—C4—C3	3.5 (6)	C27—Sn2—C23—C24	−142.4 (2)
O2—N2—C4—C5	3.2 (6)	O9—Sn2—C23—C24	105.2 (2)
O1—N2—C4—C5	−177.9 (4)	O7—Sn2—C23—C24	−64.9 (3)
C3—C4—C5—C6	0.4 (5)	Sn2^i^—Sn2—C23—C24	68.3 (3)
N2—C4—C5—C6	−178.2 (3)	Sn2—C23—C24—C25	178.1 (2)
C4—C5—C6—C1	0.7 (5)	C23—C24—C25—C26	−173.1 (3)
C4—C5—C6—C7	−179.1 (3)	O9^i^—Sn2—C27—C28	−28.8 (3)
C2—C1—C6—C5	−0.8 (5)	C23—Sn2—C27—C28	140.8 (3)
C2—C1—C6—C7	179.0 (3)	O9—Sn2—C27—C28	−107.1 (2)
Sn1—O3—C7—O4	12.7 (4)	O7—Sn2—C27—C28	62.5 (2)
Sn1—O3—C7—C6	−164.9 (2)	Sn2^i^—Sn2—C27—C28	−70.3 (2)
C5—C6—C7—O4	−12.8 (5)	Sn2—C27—C28—C29	−177.6 (2)
C1—C6—C7—O4	167.4 (3)	C27—C28—C29—C30	−68.9 (4)

Symmetry codes: (i) −*x*, −*y*+1, −*z*.

### Hydrogen-bond geometry (Å, °)

**Table:**

Cg1 is the centroid of the C16–C21 phenyl ring.

**Table:**

*D*—H···*A*	*D*—H	H···*A*	*D*···*A*	*D*—H···*A*
N1—H1N1···O6	0.91	2.59	3.422 (4)	153
N1—H2N1···O1	1.03	1.83	2.644 (5)	133
N3—H1N3···O4^ii^	0.88	2.04	2.910 (4)	167
N3—H2N3···O5	0.88	2.06	2.669 (4)	125
C17—H17A···O4^ii^	0.93	2.51	3.246 (5)	137
C30—H30A···Cg1^iii^	0.96	2.80	3.584 (5)	139

Symmetry codes: (ii) *x*+1, *y*, *z*−1; (iii) −*x*+1, −*y*+1, −*z*.
